# ZEB1-AS1 and miR-200b-3p Expression in Non-Muscle-Invasive Bladder Cancer: Pathological Correlates

**DOI:** 10.3390/jcm15114182

**Published:** 2026-05-28

**Authors:** Enes Degirmenci, Oner Sanli, Murat Kaytaz, Canan Kucukgergin, Selcuk Erdem, Sule Seckin, Yasemin Ozluk, Faruk Ozcan

**Affiliations:** 1 Department of Urology, Istanbul Faculty of Medicine, Istanbul University, Istanbul 34093, Turkey; 2 Division of Urologic Oncology, Department of Urology, Istanbul Faculty of Medicine, Istanbul University, Istanbul 34093, Turkey; onersanli@hotmail.com (O.S.);; 3 Department of Medical Biochemistry, Istanbul Faculty of Medicine, Istanbul University, Istanbul 34093, Turkey; 4 Department of Pathology, Istanbul Faculty of Medicine, Istanbul University, Istanbul 34093, Turkey

**Keywords:** biomarkers, bladder cancer, long non-coding RNA, microRNA, non-muscle-invasive bladder cancer

## Abstract

**Purpose:** Long non-coding RNAs (lncRNAs) and microRNAs (miRNAs) have emerged as important regulators of the epithelial–mesenchymal transition (EMT) and tumor progression. The present study evaluated the expression profiles of lncRNA ZEB1-AS1 and miR-200b-3p in non-muscle-invasive bladder cancer (NMIBC) tissues and investigated their associations with clinicopathological characteristics. **Materials and Methods:** Tumor tissues and matched adjacent normal bladder tissue were obtained from 50 patients with primary NMIBC who underwent transurethral resection of bladder tumors. Total RNA was extracted, and expression levels of ZEB1-AS1 and miR-200b-3p were measured using quantitative real-time polymerase chain reaction (qRT-PCR). Relative expression levels were calculated using the 2^−ΔΔCT^ method. Associations between gene expression levels and clinicopathological parameters were analyzed using non-parametric statistical tests. Receiver operating characteristic (ROC) curve analysis was performed to evaluate diagnostic performance. **Results:** ZEB1-AS1 and miR-200b-3p demonstrated significantly increased expression in tumor tissues compared with adjacent normal bladder tissue (*p* = 0.018 and *p* = 0.034, respectively). ZEB1-AS1 expression was significantly higher in high-grade tumors (*p* = 0.007), whereas miR-200b-3p expression was more pronounced in low-grade tumors (*p* = 0.015). No significant associations were identified between expression levels and tumor stage, carcinoma in situ, or variant histology. ROC analysis demonstrated modest diagnostic performance, with AUC values of 0.637 for ZEB1-AS1 and 0.624 for miR-200b-3p. **Conclusions:** ZEB1-AS1 and miR-200b-3p demonstrated distinct expression patterns associated with tumor grade and may contribute to EMT-associated molecular heterogeneity in NMIBC. Although the individual diagnostic performance of these markers appeared limited, the present findings provide additional insight into non-coding RNA-associated pathways and support further investigation in larger validation studies.

## 1. Introduction

Bladder cancer (BC) represents a major malignancy of the urinary tract and remains an important global health burden [1]. Non-muscle-invasive bladder cancer (NMIBC), which includes stages Ta, T1, and carcinoma in situ (CIS), makes up about 75% of newly diagnosed BC cases. Although NMIBC generally has a more favorable prognosis than muscle-invasive disease, it is characterized by a high recurrence rate of up to 70% and a 10–30% risk of progression to muscle-invasive BC [2,3]. NMIBC requires long-term surveillance because of its high recurrence potential, making it one of the most costly malignancies to manage on a per-patient basis. Although cystoscopy remains the gold standard for diagnosis and surveillance, its invasive nature and limited sensitivity for certain lesions have stimulated increasing interest in molecular biomarker development [4].

Recent studies have increasingly focused on integrating non-invasive molecular biomarkers, including urinary and tissue-based markers, with or as alternatives to conventional cystoscopy, aiming to enhance diagnostic accuracy, reduce invasiveness, and improve surveillance strategies in bladder cancer [4]. In particular, non-coding RNAs (ncRNAs) have emerged as attractive biomarker candidates because of their relative stability in tissue and body fluids, including urine and plasma, making them suitable for non-invasive molecular testing approaches [5,6]. Beyond their diagnostic potential, ncRNAs also function as key regulators of gene expression by influencing transcriptional, post-transcriptional, and epigenetic regulatory networks. Long non-coding RNAs (lncRNAs), in particular, have been implicated in tumor initiation, progression, and therapeutic resistance through interactions with chromatin, transcription factors, and microRNAs [7,8].

Among ncRNA-associated pathways implicated in bladder cancer, EMT-related regulatory networks have attracted particular attention due to their potential association with tumor aggressiveness and disease progression [9]. Within EMT-associated regulatory networks, ZEB1-AS1 is of particular interest because it is a natural antisense transcript of the ZEB1 gene, a central transcriptional regulator involved in epithelial–mesenchymal transition. Previous studies have suggested that ZEB1-AS1 may contribute to bladder cancer progression by promoting cell proliferation, migration, invasion, and EMT-associated phenotypic changes [9,10]. Mechanistically, ZEB1-AS1 has been reported to function as a competing endogenous RNA (ceRNA), sequestering members of the miR-200 family and thereby modulating miR-200/ZEB-mediated regulatory networks [9,11]. This axis is especially relevant in NMIBC, where EMT-related molecular alterations may contribute to biological heterogeneity, tumor recurrence, intravesical dissemination, and progression to muscle-invasive disease [12]. In contrast, the miR-200 family, particularly miR-200b-3p, is widely recognized as an important regulator of epithelial differentiation and EMT suppression. miR-200b-3p can directly target ZEB1 and ZEB2 transcripts, thereby helping to maintain epithelial characteristics and potentially limiting tumor aggressiveness [13,14]. Therefore, the simultaneous evaluation of ZEB1-AS1 and miR-200b-3p may provide a more integrated understanding of EMT-associated non-coding RNA regulation in NMIBC than evaluating either marker alone. However, interactions between lncRNAs and miRNAs are highly dynamic and context-dependent, and the simultaneous dysregulation of oncogenic and tumor-suppressive ncRNAs has been described in multiple cancer types, reflecting the complexity of ceRNA-mediated regulatory networks [15,16].

Despite these advances, data regarding the co-expression patterns of ZEB1-AS1 and miR-200b-3p and their clinicopathological significance in NMIBC remain limited and partially inconsistent across studies [9,10]. Accordingly, the present study aimed to evaluate the expression levels of ZEB1-AS1 and miR-200b-3p in NMIBC tissues and to investigate their associations with clinicopathological features, including tumor grade and stage. Their potential diagnostic utility was additionally explored using receiver operating characteristic (ROC) curve analysis. By characterizing the expression profiles of these non-coding RNAs in NMIBC, this study may contribute to a more refined understanding of EMT-associated molecular alterations in bladder cancer. Given the limited availability of NMIBC-specific expression data and the biological complexity of EMT-associated pathways, additional observational studies may help further clarify these molecular interactions and support future biomarker-oriented research.

## 2. Materials and Methods

### 2.1. Patient Selection and Tissue Sampling

The present study protocol was reviewed and approved by the Institutional Review Board of Istanbul University, Istanbul Faculty of Medicine (approval number: 913411; approval date: 8 June 2022). Written informed consent was obtained from all participants at the time of enrollment. The study was conducted in accordance with the principles of the Declaration of Helsinki. Fifty patients with primary NMIBC who underwent transurethral resection of bladder tumor (TUR-BT) between 5 July 2022 and 30 March 2023 were prospectively enrolled in the study. Tumor tissues and matched adjacent normal bladder tissue were collected during surgery. Adjacent tissue samples were obtained from macroscopically normal bladder mucosa located at least 3 cm from the tumor margin and were confirmed to be free of tumor involvement by pathological evaluation prior to molecular analysis. None of the patients had received prior intravesical therapy, chemotherapy, or radiotherapy.

### 2.2. RNA Extraction and Quantification

Total RNA was extracted from tissue samples using the miRNeasy Tissue/Cells Advanced Kit (Qiagen, Hilden, Germany, Cat. No. 217684) according to the manufacturer’s instructions. Tissue samples were initially added to the supplied lysis buffer and homogenized prior to RNA isolation. RNA quantity and purity were evaluated spectrophotometrically using NanoDrop (Thermo Fisher Scientific, Wilmington, DE, USA). Samples with A260/A280 ratios between 1.8 and 2.0 were considered acceptable for downstream analyses. RNA concentrations were measured and recorded for each sample. Complementary DNA (cDNA) synthesis for lncRNA and miRNA analyses was performed using the RT^2^ First Strand Kit (Qiagen, Hilden, Germany, Cat. No. 339340) and the miRCURY LNA RT Kit (Qiagen, Hilden, Germany, Cat. No. 339346), respectively. Synthesized cDNA samples were stored at −20 °C until further analysis.

### 2.3. Quantitative Real-Time Polymerase Chain Reaction Analysis

Quantitative real-time polymerase chain reaction (qRT-PCR) analyses were performed using a Rotor-Gene Q platform (Qiagen, Hilden, Germany). The following assays were used in the study: GAPDH endogenous control (Qiagen, Hilden, Germany, Cat. No. LPH31725A); RT^2^ lncRNA qPCR Assay for ZEB1-AS1 (Qiagen, Hilden, Germany, Cat. No. LPH12834A); miRCURY LNA™ miRNA PCR Assay for hsa-miR-200b-3p (Qiagen, Hilden, Germany, Cat. No. YP00206071); miRCURY LNA™ miRNA PCR Assay for U6 snRNA (Qiagen, Hilden, Germany, Cat. No. YP00203907). An lncRNA expression analysis was performed using the RT^2^ lncRNA qPCR Assay Kit (Qiagen, Hilden, Germany) with an initial denaturation at 95 °C for 10 min, followed by 40 cycles of 95 °C for 15 s and 60 °C for 30 s. A miRNA expression analysis was performed using the miRCURY LNA™ miRNA PCR Assay Kit (Qiagen, Hilden, Germany) with an initial denaturation step at 95 °C for 2 min, followed by 40 cycles of 95 °C for 10 s and 56 °C for 60 s. GAPDH and U6 snRNA were used as endogenous reference genes for the normalization of lncRNA and miRNA expression levels, respectively. All qRT-PCR reactions were performed in triplicate. Relative expression levels were determined using the 2^−ΔΔCT^ method and expressed as fold-change values relative to adjacent normal bladder tissues. ΔΔCT values were calculated as follows: (CT value of the target ncRNA in tumor tissue − CT value of the endogenous reference ncRNA in tumor tissue) − (CT value of the target ncRNA in normal tissue − CT value of the endogenous reference ncRNA in normal tissue). Therefore, summarized fold-change values do not necessarily correspond directly to ratios derived from group median expression values.

### 2.4. Statistical Analysis

Statistical analyses and data visualization were performed using SPSS version 26.0 (IBM Corp., Armonk, NY, USA). Continuous variables were summarized as median values with interquartile ranges (IQRs), whereas categorical variables were expressed as frequencies and percentages. The normality of data distribution was assessed using the Shapiro–Wilk test. Since expression data were not normally distributed, non-parametric statistical methods were applied. Comparisons of expression levels between tumor and adjacent normal bladder tissue were performed using the Wilcoxon signed-rank test. Differences in expression levels according to clinicopathological variables were analyzed using the Mann–Whitney U test or Kruskal–Wallis test, as appropriate. Correlations between variables were evaluated using Spearman’s rank correlation analysis. Receiver operating characteristic (ROC) curve analysis was performed to evaluate the discriminatory performance of ZEB1-AS1 and miR-200b-3p expression levels in distinguishing tumor tissues from adjacent normal bladder tissue. The area under the curve (AUC) was calculated to assess diagnostic performance. All statistical tests were two-sided, and a *p*-value < 0.05 was considered statistically significant.

## 3. Results

### 3.1. Patient Characteristics

The baseline demographic and clinicopathological characteristics of the study population are summarized in Table 1.

The study involved 50 patients diagnosed with primary NMIBC. The median age was 69 years (interquartile range [IQR], 59–78 years), and 48 patients (96%) were male.

Regarding tumor characteristics, 30 patients (60%) had Ta tumors and 20 (40%) had T1 tumors. Low-grade tumors were observed in 21 patients (42%), while 29 patients (58%) had high-grade disease. Concomitant carcinoma in situ (CIS) was present in 13 cases (26%), and variant histology was identified in three cases (6%).

### 3.2. Expression Levels of ZEB1-AS1 and miR-200b-3p

The expression levels of ZEB1-AS1 and miR-200b-3p in tumor and adjacent normal bladder tissue are presented in Table 2.

**Figure 1:**
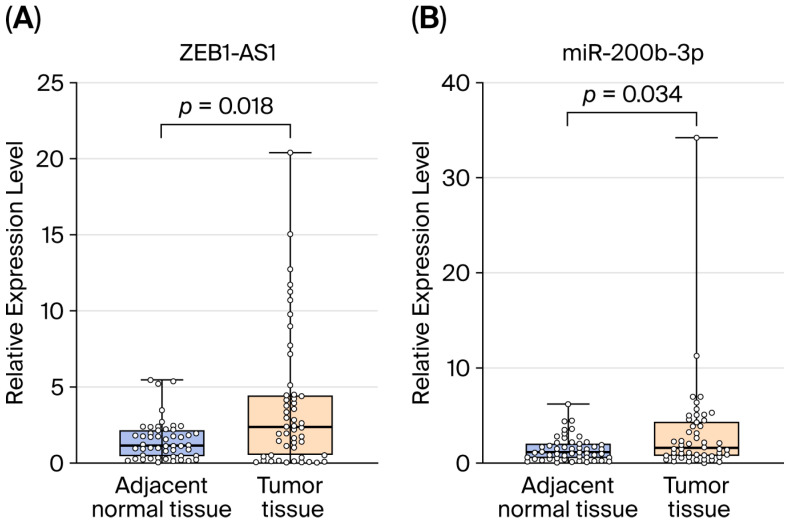
Relative expression levels of ZEB1-AS1 (**A**) and miR-200b-3p (**B**) in NMIBC tumor tissues and adjacent normal bladder tissue. Data are presented as median values with interquartile ranges.

ZEB1-AS1 and miR-200b-3p expression levels were significantly higher in tumor tissues compared with adjacent normal bladder tissue (*p* = 0.018 and *p* = 0.034, respectively) (Figure 1A,B).

### 3.3. Correlation with Clinicopathological Features

The relationship between gene expression levels and tumor stage is shown in Table 3.

**Figure 2:**
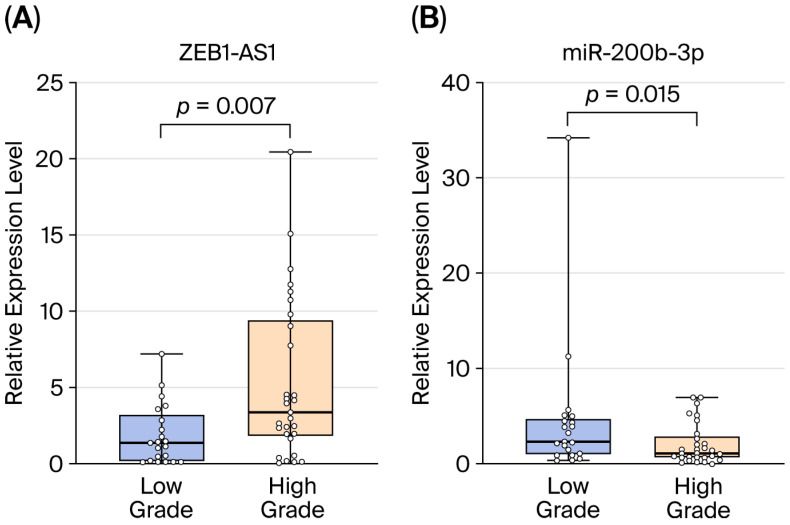
Expression levels of ZEB1-AS1 (**A**) and miR-200b-3p (**B**) according to tumor grade in NMIBC.

ZEB1-AS1 expression was significantly higher in high-grade tumors compared with low-grade tumors (*p* = 0.007) (Figure 2A). In contrast, miR-200b-3p expression was significantly higher in low-grade tumors (*p* = 0.015) (Figure 2B).

No significant associations were identified between ZEB1-AS1 or miR-200b-3p expression levels and clinical parameters, including age, smoking status, and body mass index; or pathological features, including the presence of CIS and variant histology (all *p* > 0.05).

### 3.4. ROC Curve Analysis

**Figure 3:**
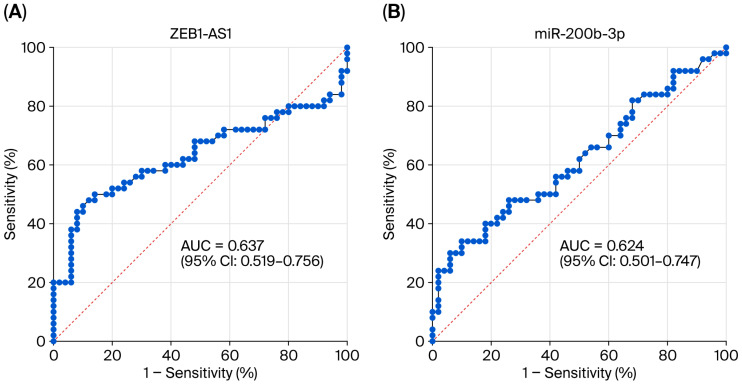
Receiver operating characteristic (ROC) curve analysis of ZEB1-AS1 (**A**) and miR-200b-3p (**B**) for distinguishing NMIBC tumor tissues from adjacent normal bladder tissue.

The receiver operating characteristic (ROC) analysis demonstrated the modest diagnostic performance of both markers in distinguishing tumor tissues from adjacent normal bladder tissue. The AUC was 0.637 (95% CI: 0.519–0.756) for ZEB1-AS1 and 0.624 (95% CI: 0.501–0.747) for miR-200b-3p (Figure 3A,B).

## 4. Discussion

BC is a molecularly heterogeneous malignancy characterized by substantial variability in recurrence patterns, progression risk, and therapeutic response [17]. Despite advances in risk stratification and surveillance strategies, the management of NMIBC remains challenging because of its high recurrence potential and the requirement for prolonged follow-up. Current surveillance approaches primarily rely on cystoscopy and urine cytology; however, these methods remain associated with important limitations, including invasiveness, patient burden, and reduced sensitivity in selected clinical settings, particularly for low-grade lesions [18]. Consequently, considerable attention has been directed toward the identification of molecular biomarkers capable of improving disease characterization and patient stratification. In recent years, non-coding RNAs have emerged as promising biomarker candidates because of their regulatory roles in tumor biology and their potential utility in tissue- and urine-based diagnostic platforms [6].

In the present study, we investigated the expression profiles of ZEB1-AS1 and miR-200b-3p in NMIBC tissues and evaluated their associations with clinicopathological characteristics. Both markers demonstrated significantly increased expression in tumor tissues relative to adjacent normal bladder tissue. Furthermore, ZEB1-AS1 expression was significantly elevated in high-grade tumors, whereas miR-200b-3p expression was relatively more pronounced in low-grade lesions. These findings may be compatible with EMT-associated molecular heterogeneity in NMIBC; however, the underlying biological mechanisms remain to be clarified in future functional studies.

The epithelial–mesenchymal transition (EMT) represents a key biological process involved in tumor progression, invasion, metastatic dissemination, and therapeutic resistance [13,19]. Previous studies have demonstrated that the activation of EMT-associated pathways contributes not only to invasive behavior but also to phenotypic plasticity and disease progression in bladder cancer. Within this framework, interactions between long non-coding RNAs and microRNAs have attracted increasing attention because of their capacity to regulate multiple downstream signaling pathways. In particular, competing endogenous RNA (ceRNA) networks may influence tumor biology through the modulation of miRNA activity and EMT-related signaling cascades [11,15].

Among EMT-associated lncRNAs, ZEB1-AS1 has increasingly emerged as an oncogenic regulator implicated in multiple malignancies, including bladder cancer. Previous investigations demonstrated that ZEB1-AS1 may promote cell proliferation, migration, invasion, and EMT-associated phenotypic alterations [9,10]. Gao et al. [9] further reported that ZEB1-AS1 may facilitate bladder cancer progression through the modulation of the miR-200b/FSCN1 signaling pathway, supporting its role in EMT-associated regulatory networks. Similarly, Lin et al. [10] observed associations between elevated ZEB1-AS1 expression and adverse clinicopathological characteristics, including higher histological grade and advanced disease stage. Our findings of significantly increased ZEB1-AS1 expression in high-grade NMIBC are therefore consistent with a possible association between ZEB1-AS1 upregulation and adverse clinicopathological characteristics.

The biological role of miR-200 family members appears more complex. The miR-200 family has been widely recognized as an important regulator of epithelial differentiation and EMT suppression through the direct targeting of ZEB-associated pathways [13,14]. Previous meta-analytic evidence further suggested that elevated miR-200 family expression may be associated with favorable oncological outcomes and the suppression of invasive phenotypes [20]. However, the expression profiles of miR-200 family members in bladder cancer have demonstrated heterogeneous findings across studies. While several investigations reported reduced expression in invasive disease, others observed increased expression within tumor tissues [9,13]. Such discrepancies may arise from differences in patient populations, disease stage, analytical methodology, and biological heterogeneity. Since our cohort consisted exclusively of NMIBC patients, the observed expression patterns may reflect disease-stage-specific molecular characteristics rather than universal expression profiles across all bladder cancer subtypes.

No significant associations were identified between expression levels and tumor stage, concomitant CIS, or variant histology. However, the interpretation of these findings requires caution because subgroup analyses were limited by relatively small patient numbers, particularly for CIS and variant histology groups. Therefore, the absence of statistically significant associations should not necessarily be interpreted as evidence of biological independence, and larger multicenter investigations are required to further clarify these relationships.

From a translational perspective, ROC analysis yielded AUC values of 0.637 for ZEB1-AS1 and 0.624 for miR-200b-3p. While these findings indicate relatively limited discriminative ability, the markers may still contribute to multimodal or combined biomarker approaches. Accordingly, the present findings do not support the immediate clinical implementation of either biomarker individually. However, previous studies suggest that molecular biomarkers frequently demonstrate improved predictive performance when incorporated into multimarker panels rather than assessed in isolation [18,21,22]. Therefore, although exploratory, these findings may support future investigations integrating broader biomarker combinations with clinicopathological variables. Recent exploratory investigations have similarly highlighted the growing interest in emerging non-invasive urinary and molecular biomarkers for NMIBC surveillance, risk stratification, and diagnostic support. Collectively, these studies further suggest that molecular biomarkers may provide greater clinical utility when incorporated into multimarker or complementary surveillance strategies rather than being applied as standalone diagnostic tools [23,24,25].

A methodological strength of the present study was the use of paired tumor and adjacent normal bladder tissue samples, allowing intra-patient comparison while reducing interindividual biological variability. Adjacent tissue samples were obtained from macroscopically normal bladder mucosa and underwent pathological confirmation prior to molecular analyses. However, despite pathological confirmation, the use of adjacent tissue controls may still be influenced by potential field cancerization effects in bladder cancer, which should be considered when interpreting tumor–control comparisons. Therefore, future studies incorporating additional molecular characterization approaches may further strengthen control tissue selection and reduce potential biological heterogeneity.

Several limitations of this study should be acknowledged. First, independent external validation cohorts and publicly available transcriptomic datasets were not incorporated, limiting the generalizability of the findings. Second, the absence of longitudinal follow-up data precluded evaluation of recurrence- and progression-related outcomes. In addition, the limited sample size precluded the application of more robust statistical approaches, including multivariable analyses. Furthermore, the predominantly male composition of the cohort limited the assessment of potential sex-related molecular variability.

Finally, as no functional validation experiments were performed, the biological mechanisms potentially underlying the ZEB1-AS1/miR-200b-3p axis remain to be further elucidated in future mechanistic studies. Despite these limitations, the present study provides further insight into EMT-associated non-coding RNA expression patterns in NMIBC and contributes to the growing understanding of molecular pathways potentially involved in bladder cancer biology. Future multicenter studies incorporating external validation, longitudinal outcomes, and functional analyses are warranted to clarify the biological and potential clinical significance of the ZEB1-AS1/miR-200b-3p regulatory axis.

## 5. Conclusions

This study demonstrated altered expression patterns of ZEB1-AS1 and miR-200b-3p in NMIBC and identified associations between these EMT-associated non-coding RNAs and selected clinicopathological characteristics. ZEB1-AS1 expression was significantly associated with high-grade disease, whereas miR-200b-3p showed distinct expression patterns across tumor subgroups, suggesting potential involvement in NMIBC biological heterogeneity. Although the diagnostic performance of these markers alone appeared modest, the findings contribute to the growing understanding of EMT-related molecular pathways in bladder cancer and may support future investigations evaluating their role within broader biomarker strategies. Further multicenter studies incorporating longitudinal outcomes, external validation cohorts, and functional analyses are warranted to clarify the biological and translational significance of the ZEB1-AS1/miR-200b-3p axis.

## Figures and Tables

**Table 1:** Baseline clinicopathological characteristics of the study population.

Variable	Overall (*n* = 50)
Age (y)	69 (59–78)
BMI (kg/m^2^)	27.18 (24.91–29.05)
Gender	
Male	48 (96%)
Female	2 (4%)
Smoking history	
Non-smoker	6 (12%)
Smoker	44 (88%)
Pack-years in smokers	40 (29–50)
Chief complaint	
Microhematuria	3 (6%)
Macrohematuria	33 (66%)
LUTS ^a^	7 (14%)
Asymptomatic	7 (14%)
Tumor stage	
Ta	30 (60%)
T1	20 (40%)
Tumor grade	
Low grade	21 (42%)
High grade	29 (58%)
Presence of CIS	
Absent	37 (74%)
Present	13 (26%)
Variant histology	
Absent	47 (94%)
Present	3 (6%)

^a^ LUTS, lower urinary tract symptom. Data are presented as median (interquartile range) or number (%).

**Table 2:** Expression levels of ZEB1-AS1 and miR-200b-3p in tumor and adjacent normal bladder tissue.

Gene	Tissue	*n*	Median (IQR)	*p*-Value
ZEB1-AS1	Bladder tumor	50	2.38 (0.54–4.46)	0.018
	Adjacent normal bladder tissue	50	1.16 (0.46–2.11)	
miR-200b-3p	Bladder tumor	50	1.69 (0.82–3.91)	0.034
	Adjacent normal bladder tissue	50	1.15 (0.55–2.01)	

Data are presented as median (interquartile range).

**Table 3:** Expression levels of ZEB1-AS1 and miR-200b-3p according to tumor stage.

Gene	Stage	*n*	Median (IQR)	*p*-Value
ZEB1-AS1	Ta	30	1.71 (0.05–11.72)	0.235
	T1	20	2.82 (0.06–20.41)	
miR-200b-3p	Ta	30	2.24 (0.02–34.20)	0.230
	T1	20	1.41 (0.16–6.99)	

## Data Availability

The data presented in this study are available on reasonable request from the corresponding author.
